# Do we have friendly services to meet the needs of young women exposed to intimate partner violence in the Madrid region?

**DOI:** 10.1111/hex.13453

**Published:** 2022-02-24

**Authors:** Eva Durán‐Martín, Carmen Vives‐Cases, Laura Otero‐García, Esther Castellanos‐Torres, Belen Sanz‐Barbero

**Affiliations:** ^1^ International Doctoral School Universidad Nacional de Estudios a Distancia (UNED) Madrid Spain; ^2^ Department of Community Nursing, Preventive Medicine and Public Health and the History of Science Universitat d' Alacant, CIBER of Epidemiology and Public Health (CIBERESP) Madrid Spain; ^3^ Department of Nursing Universdad Autónoma de Madrid, CIBER of Epidemiology and Public Health (CIBERESP) Madrid Spain; ^4^ Department of Social Sciences Universidad Carlos III de Madrid Madrid Spain; ^5^ Department of Epidemiology and Biostatistics Institute of Health Carlos III, CIBER of Epidemiology and Public Health (CIBERESP) Madrid Spain

**Keywords:** friendly services, intimate partner violence, young women

## Abstract

**Introduction:**

Women experiencing intimate partner violence (IPV) do not tend to go very frequently to formal support services. The objective of this study is to identify barriers related to the accessibility, acceptability, equity, appropriateness and effectiveness of IPV services from the perspective of the professionals working in the IPV public services.

**Methods:**

A qualitative study was carried out in the Madrid region based on 13 semi‐structured interviews of young women who had survived IPV as well as 17 interviews with professionals. A thematic content analysis was performed, guided by the dimensions proposed by the World Health Organization (WHO) for friendly services for young people.

**Results:**

From the perspective of the young women and professionals, barriers were identified for all the dimensions of the WHO's friendly services for young people: accessibility: lack of information and support from the social setting, scarce dissemination of the services, economic cost, non‐adapted schedules, inadequate locations or lack of services in settings close to young people; acceptability: lack of protocols to guarantee confidentiality, lack of speed in the provision of services or their referral, unwelcoming environments or unsympathetic professional malpractice; equity: discriminatory professional attitudes towards groups with different social status and lack of protocols to ensure the care of these groups; appropriateness: unmet needs and lack of multidisciplinary teams; and effectiveness: shortage of time, resources, competent professionals, protocols and coordination.

**Conclusions:**

Strategies are needed to make the necessary changes to promote friendly services for the care of young people exposed to IPV. Additionally, it must be emphasized that resources are needed to raise awareness and disseminate IPV services, as well as to train professionals in this area.

**Patient or Public Contribution:**

This paper is based on professionals' perspectives of public IPV‐related services of different areas such as Psychology, Social Work, Nursing, Psychiatry, Social Education and young women exposed to IPV. They either work in the public administration at the local, regional or state level or in NGOs in Spain.

## 1 INTRODUCTION

The World Health Organization (WHO) recognizes intimate partner violence (IPV) as one of the most common forms of violence against women. It includes any behaviour of the partner or ex‐partner that causes physical, sexual and/or psychological harm, including physical aggression, emotional abuse, sexual coercion and controlling behaviours.^1^ IPV causes short‐, medium‐ and long‐term impacts on both the physical and psychological health of women exposed to this public health problem.^2^

The characteristics of women and their social circumstances play a role in IPV and mortality risk due to IPV.^3^, ^4^ In this sense, young women and teens belong to groups that are especially vulnerable to IPV.^5^, ^6^, ^7^, ^8^, ^9^ In Spain, 19.3% of women between the ages of 16 and 24 years have, at some point in their life, suffered from physical and/or sexual IPV, and 46.1% have suffered from psychological IPV.^10^

The strategies used in seeking help when faced with an IPV situation may also differ depending on the age of the women,^11^, ^12^ with the youngest being the ones who tend to less frequently seek out professionals and formal support services, such as healthcare services, social and legal support services, or even associations.^12^ When faced with IPV, young women tend to ask the people they trust from their immediate environment for help.^12^

With the aim of bringing social and healthcare services closer to the youth population, the WHO proposed a model with basic dimensions that characterized the friendly services for young people.^13^ This model was developed in the sexual and reproductive health field due to the beneficial impact that its use could have on the health of young people. This model assumes that, for a service to be friendly, it must be: (1) accessible: young people are able to use the social and healthcare services that are available to them; (2) acceptable: young people want to and are willing to use such services; (3) equitable: all young people have the services available to them regardless of their social status/condition; (4) appropriate: services provide everything that young people may need; and (5) effective: services are adequately provided, making a positive contribution to health.^13^

In Spain, primary care provides many opportunities to implement friendly services for young people,^14^ although a transformation is needed to improve the fulfilment of young people's needs.^14^, ^15^ One of these needs is tackling youth IPV, which should be a priority not only in primary care but also in the rest of the social health services that aim to provide a response to this problem. For this reason, it is necessary to bring services closer to young women suffering from this violence situation, given the scant use that they make of the services available for this purpose.^12^

The aim of this study is to identify barriers related to the accessibility, acceptability, equity, adequacy and effectiveness of IPV services for young women in the Madrid region, from their own perspective and that of the professionals working in the public services set up to respond to the IPV problem.

## 2 METHODS

This study includes 13 semi‐structured interviews with young women who have affected by intimate partner violence (Table 1) and 17 interviews with professionals (14 women and 3 men). These professionals work with IPV situations in different areas such as Psychology, Social Work, Nursing, Psychiatry and Social Education. They either work in the public administration at the local, regional or state level or in NGOs (Table 2). This study was carried out in the Madrid region, which includes the capital of Spain and surrounding towns, with a large rural area. It is worth noting that in 2019, 15% (*n* = 163,772) of registered women in the whole region were between 15 and 29 years old; among these women, 16.7% (27,320) were not born in Spain.^16^

**Table 1:** Characteristics of the women interviewed

Young women	Sociodemographic data
YW1	36 years old. San Fernando de Henares. High school studies. Employed. Lives with her mother and sister. No children.
YW2	35 years old. Madrid. Bachelor degree studies. Employed. Lives alone. No children.
YW3	27 years old. Fuenlabrada. Completed high school. Employed. Lives alone. No children.
YW4	19 years old. Fuenlabrada. High school studies. Unemployed. Lives with her family. No children.
YW5	16 years old. Fuenlabrada. Student. Lives with her mother. No children.
YW6	22 years old. Daganzo de Arriba. Higher‐level technical/vocational school. Employed. Lives with her family. No children.
YW7	24 years old. Torres de la Alameda y Torrejón de Ardoz. Completed secondary education. Unemployed. Vive con su padre. No children.
YW8	18 years old. Daganzo de Arriba. Completed secondary education. Unemployed. Lives with her family. No children.
YW9	29 years old. Madrid. Master's degree. Student visa that only allows practical training. Shares flat with friends. No children.
YW10	22 years old. Tielmes. Completed secondary education. Employed. Lives alone. No children.
YW11	27 years old. Madrid. Completed High School. Employed. Lives with her daughter and her mother.
YW12	27 years old. Campo real. Higher‐level technical/vocational school in aesthetics and beauty. Unemployed, wanting to set up a business. Lives alone with her 6‐year‐old son.
YW13	21 years old. Madrid (Tetuán). Integration cycle. Works and studies. Lives alone in a rented property. No children.

*Note*: Community of Madrid, 2019.

**Table 2:** Characteristics of the professionals interviewed

Sex	Area or professional profile	Scope of action of the service
M	VIOGEN professional	State
M	VIOGEN professional	State
M	VIOGEN professional	State
F	Nurse responsible for IPV in a health centre	Autonomic/municipal
F	Psychiatrist in a mental health centre	Autonomic/municipal
F	Social worker in a mental health centre	Autonomic/municipal
F	Psychologist in a specific service of sexual violence	Autonomic/association
F	Psychologist in a specific service of youth violence	Autonomic/association
F	Social worker in a specific violence centre of the Autonomous Region of Madrid (CAM)	Autonomic/association
F	Social worker in a local corporation	Municipal
F	Social worker and psychologist in a local corporation. Municipal point of gender violence (PMVG) in a municipality of the CAM	Municipal
F	Social worker of PMVG in a municipality of the CAM	Municipal
F	Responsible for the youth area in a local corporation	Municipal
F	Responsible for the youth area in a local corporation	Municipal
F	Nursing Management in a health centre	Municipal
F	Psychologist of a feminist association	Asociativo
F	Director of an entity related to IPV in the tertiary sector	Association

*Note*: Community of Madrid, 2019.

### 2.1 Data collection

The interview script of the professionals included topics such as their perception of the current situation of IPV in young women, their professional approach from their different settings, barriers in accessing resources for young girls suffering IPV and their proposals for improvement (Table 3). These interviews were carried out in person between March and July 2019. They were recorded and lasted between 45 and 90 min. On the other hand, the script for the interviews with the young women included topics related to their experience with IPV, how they identified they were facing IPV and their exit process. Topics related to informal and formal help resources, as well as proposals for improvement in the care of young women are also covered (Table 4). In the case of the young women, and due to the Covid‐19 pandemic, the interviews were carried out between April and September 2020 using electronic communication channels (phone call, video call or email).

**Table 3:** Summary of the script used in the interviews with professionals

1. Welcome and introduction	Contextualization; professional career; link with the IPV intervention and with the youth population
2. Current situation of IPV in young women	Opinion about IPV, specifically in a couple context in young women; differences in IPV in youth/adolescents and older women and how they influence your daily work
3. Approach to IPV from the professional practice	Perceptions regarding the knowledge about the services by young women and your response when faced with an IPV situation; perceptions on the access and use of social health services and formal resources by young women; what are the motivations of young women to make use, or not, of these type of services; contribution of the service to modify the violence situation suffered by the young woman; system for case follow‐up or implementation; assessment of other formal resources available for IPV and your work with young women
4. Difficulties in accessing the services designed to tackle IPV in young women	Main barriers for young women to access and use the formal resources for IPV; professional difficulties in effectively managing IPV in young women; perceptions regarding whether the young women find the services friendly; possible structural barriers or barriers related to training or awareness of gender equality and using an intersectional approach; service adaptations that are needed to help young women, and factors that facilitate quality care
5. Proposals for improvement	Reflection on proposals for improvement; other considerations

*Note*: Youth‐friendly intimate partner violence‐related services in the Madrid region, 2019.

Abbreviation: IPV, intimate partner violence.

**Table 4:** Script of interviews with young women

Introduction	How are you currently?; How do you feel?
Block 1	What was your process of exiting the violence like?; When did you start thinking that perhaps you were living in a violence situation?; Why do you start thinking this?; What triggered it?; How long did it take you to realize this?; When can you name it and what do you call it?
Block 2	How did the process of telling other people go?; Who did you first tell?; Why did you decide to tell that particular person?; How did they react or how did the people you leaned on react?; Then, what did you do?; what role did your family play in this process?
Block 3	How did your partner/aggressor react? (taking into account all episodes you have lived with all partners or with this partner); Formal help: What services did you seek?; what difficulties did you find in accessing the formal help?; Did you know about the help services?; How did you get the information about that service?; Did you go to any specialized service? Did they send you from one place to another?
Block 4	How did they treat you?; What did you like or not like about how you were treated? What did you expect to find, what did you find and what would you have liked to find in the help service you used?
Block 5	Did you file a complaint? (Yes); Did somebody file it for you? How did you feel in this process? Were you frightened? What role did your family have in this process? Your friends?; (No) Why did you not file a complaint?
Block 5 BIS	Only if still in a violence process. Within the Covid‐19 context, have you had to use the services during this period or interrupt the services that you were already using?
Block 6	From your experience, what would you recommend to the people who work in these services so that they are closer and friendlier for young people?
Block 7	Again, from your experience, what needs to be done to improve the care of young girls who are in an abusive situation with their boyfriend/partner and want to break up with him? Or if they have already broken up, what can they do? How can they be helped?
Closing	At this point in your life: what would you like to give yourself? What are your expectations and needs?
Sociodemographic data	Age: _______________ Living situation: Work situation:Level of studies finished: Municipality: Children:

*Note*: Youth‐friendly intimate partner violence‐related services in the Madrid region, 2019.

### 2.2 Ethical considerations

Each participant received an informed consent form, in which the objectives of the study as well as the reason for the interview were explained. Their anonymity as well as confidentiality of the opinions that they expressed were guaranteed. Furthermore, they were assured that their participation was voluntary and that they could withdraw at any time during the interviews if they so wished. The project was approved by the Ethics Committee of the Instituto de Salud Carlos III, protocol CEI PI 61_2019‐v3.

### 2.3 Analysis

The interviews were carried out using a thematic analysis based on the WHO's model of friendly services for young people.^13^ Before this, a codebook was developed to describe the operational definitions of each domain: accessible, acceptable, equitable, appropriate and effective (Table 5). Once these domains were defined, two members of the research team grouped and classified the information on barriers obtained from the interviews according to these domains. After several readings, all the authors of the manuscript debated, specified and adjusted the codes to the operational definitions.

**Table 5:** Codebook

Accessible	Free or affordable services Schedule adaptation Convenient location Knowledge about and provision of services Community support Dissemination to the community Close environment services
Acceptable	Protocol: confidentiality Appropriate setting: privacy, security and welcoming Speed of service or referral Dissemination in multiple formats Professional attitudes: good treatment Young people evaluate the services
Equitable	Protocol: equity Professional attitudes: nondiscriminatory Personnel diversity
Appropriate	Needs covered Multidisciplinary teams Holistic approach
Effective	Competent professionals Protocols and clinical practice guidelines Resources time: enough and appropriate

*Note*: Adaptation of WHO and I. Goicolea criteria about youth‐friendly health services in relation to intimate partner violence resources in the Madrid region.^13^, ^17^

## 3 RESULTS

On analysing the information, barriers were identified with regard to the accessibility, acceptability, equity, appropriateness and effectiveness of IPV services for young women in the Madrid region.

### 3.1 Accessibility

Both the professionals and the young women interviewed were in agreement in pointing out that there is a lack of information about the different services available and how to obtain the necessary help, thus making their access difficult. Many young women were unaware of the existence of the services or only knew the phone number of the information line 016. Professionals and young people feel that the lack of knowledge of the operation of the services is enhanced by the fear of the IPV situation itself. This fear stops them from asking for help or seeking other resources, as many think that they will be forced to report the situation. Likewise, there was agreement in their views that the lack of support of an IPV situation from their social environment makes it difficult for young women to seek these services. One of the young women highlights the need to provide ‘counselling for family members so that they can support women and understand the processes they could go through and how they could manage them’. This is related to the tendency of adults to deny IPV, thus having a major impact on accessibility since minors must be accompanied by a legal guardian to use these services.

Young women report that the lack of dissemination at the community level about the benefits and needs covered by the specific violence services acts as an accessibility barrier.

Finally, professionals recognized other barriers. First, although the fact that the services were not affordable or free was not pointed out as a direct barrier, a professional did highlight the economic cost (related to travel, for e.g.) and the time needed to get to the service. They also highlight the lack of adaptation to the schedules of young people or the lack of extended opening hours as a barrier to their access. Regarding where the services are located, the centralization of violence resources in the main city as well as the fact that they share spaces with social or municipal services, that is, they do not have their own space, were found to be barriers. A professional pointed out how not providing the services in settings that are close to young women, such as educational centres, is a barrier for them to obtain information that would enable them to have easier access to the services (Table 6).

**Table 6:** Barriers to accessibility to intimate partner violence‐related services for young women

Type	Barriers
Knowledge and obtaining services	‘They didn't provide me with any type of help or inform me about the help available so I didn't have any access to information and I didn't find out about the services or resources that could have helped me’ (YW7). ‘I think they don't know about it. I think they hear about it, but they don't think it's a message for them, because they do know about the 016 phone number …’ (MP4) ‘the fear…. what they're going to find there… they think they may have to file a complaint…. Perhaps they don't know that here we support them, filing a complaint is optional, we're going to respect their decision …’ (MP6). ‘Also for the fear, because I was very afraid of all of this so, no no no. I simply went to progressive women…. But then I didn't go anywhere else*’*. (YW11)
Community support	‘So, more and more, gender‐based violence is something that doesn't exist…. well it generates a lot of friction in the intervention. It's complicated to make them understand: listen, this is real, your daughter can get out of this situation, and even become stronger …’ (MP5) ‘I think one of the barriers has to do with the legal situation. You cannot ask for help until you are a certain age without your parent's consent …’ (MP15) ‘give advice to parents so they can support women and understand the processes they can go through and how to manage them’. (YW1)
Dissemination among the community	‘Most don't know how to tell their parents or families. Parents need training so they can establish healthy relationships with their daughters, so they can talk to them about this type of situations’ (YW1) ‘I think the free psychological service needs more visibility and young people need to be made aware that certain attitudes are not acceptable…’*. *(YW3)
Free or affordable services	‘The economic cost, the time, you have to miss classes. If you go to a service in Madrid and you have to be there at 2…’ (MP6).
Schedule adaptation	‘… due to the schedule, because all the appointments are taken. And another thing… a women decides… but if I decide today, I decide to take the step or file a complaint or, at least, tell somebody, I need to do it today and now…’. ‘Yes, an afternoon schedule, but not early afternoon, like until 7 or 8, because at 5 it closes and some of us can't get there at that time’. (MP6)
Convenient location	‘Being within Social Services doesn't help us. People are often confused… Social Services has its burden, its stereotype, of what people think we do and this doesn't help with what we want to do in the violence helpdesk…The rural areas are forgotten… If I want to send a teenage girl to a specific violence service for teenagers, she has to go on an outing for one and a half hours to get there and one and a half hours to get back’. (MP5); ‘…it's difficult for some girls, it's costly with respect to time, distance, etc… the services of their municipalities don't have the degree of specialisation and specificity that we have as a teenage service’. (MP14)
Services in close environments	‘…some of the teachers you have are able to open that door, you can talk to them about anything…. in an educational context this figure should always be there…. I think there are many barriers because in childhood we are neither doing the prevention correctly, nor the information….’. (MP7)

*Note*: Community of Madrid, 2019.

### 3.2 Acceptability

Young people and professionals expressed the need to have policies and procedures that guarantee the confidentiality in some of the services to make them more acceptable. Both these groups also identified barriers in the physical environment of the services, such as spaces that are not very welcoming for young people. Another barrier, on which both groups agree, is the lack of speed in providing care within the services or in referring women to other services, which makes them less acceptable to young women. Young women particularly emphasize the slowness in receiving psychological care and the lack of continuity in its provision.

On the other hand, some young women noted attitudes of professional malpractice as a barrier for finding the services acceptable and making use of them (Table 7).

**Table 7:** Barriers to acceptability of intimate partner violence‐related services for young women

Types	Barriers
Protocol: confidentiality	‘… the official who attended me did not have the perspective of keeping my situation confidential… she spoke out loud so the rest of the people around would know that I was requesting help for gender‐based violence’. (YW1) ‘During these years I've managed complaints where I have had the victim here and, behind them, there was another boy picking up a complaint for loss of ID, robbery by force, or sometimes people or arrestees, just walking behind., a disaster. So apart from improving that as much as possible… informing them’. (HP16)
Adequate setting: privacy, safe and welcoming	‘I also didn't like the facilities. They reminded me of social services, they gave me negative connotations…’ (YW2) ‘in the first place, I think they think this is a service for adults… we have to adapt the messages…. make them feel more comfortable coming to an office like this one…. intervene in a much more informal manner’. (MP5)
Speed in the provision of services or referrals	‘I tried to see psychologists through the social security, but they gave me appointments every 3 months’ (YW3); ‘they told me not to file the complaint that day. I had to go three times because they didn't believe me or they didn't think it was that serious. Also, they take ages to give you an appointment with a psychologist’. (YW4); ‘The social services of the area, that doesn't work…. they have a very long waiting list’. (MP13)
Professional malpractice attitudes	‘And in the hospital, the doctor that treated me told me that it was my fault for being with him’ (YW4); ‘I think everyone should doubt things, whoever says them, but even so you need to treat them well… With the judge ruling, they made me believe it wasn't that bad and that I was lying…’ (YW4); ‘What I didn't like was the reply of a policeman’. He told me ‘be very careful with what you say, whatever you say now is going to mark his record for life’ he told me ‘be careful, don't invent things’ something like that, and of course, I was astonished. Me, who hadn't done anything and that at last I had decided to ask the police for help…’. (YW12)

*Note*: Community of Madrid, 2019.

### 3.3 Equity

Professionals and young women agree that the discriminatory and stereotyped attitudes in the professional treatment of women pose an important barrier, particularly for the care of minors, women with disabilities or immigrant women.

The professionals highlight the need to have protocols to ensure equitable provision of services to young women and also the vulnerable groups mentioned previously. In the case of immigrant women or ethnic minorities, such as the gypsy ethnic group, the professionals agree that these women are more vulnerable due to barriers such as language, the social prejudices of the professionals, such as racism, and the lack of support in their social environment (Table 8).

**Table 8:** Equity barriers in intimate partner violence‐related services for young women

Types	Barriers
Discriminatory professional attitudes	‘…in vulnerable populations there is a lot of attention bias because, for example …with the gypsies (that's what colleagues say) you can't do anything, because you're risking your life because male gypsies are sooo gypsy, so… they are left aside’ (MP13). ‘I didn't like the treatment…’ on the other hand, they made racist comments… like I don't know… the superior nun came and told me ‘don't leave any stains because in your house you didn't have a floor…’ and ‘these things don't happen in your country’ or ‘it's worse in your country…’ (YW10)
Protocol: equity	‘…there is an intersectionality problem in the services. As soon as a situation pops up, a condition that makes you more vulnerable, the services aren't ready for that nor are they oriented to that group… there are no policies targeting young people in general….’ (MP9); ‘there is a situation of maximum vulnerability with immigrants, that of course, many times the language, many times… there are lots of problems…. … (MP2)’; ‘…when immigrant women see that they have a system that protects them, they ask for help and they leave…, they are over‐represented in the network and in the residential resources, also because when they do decide to leave they have a much smaller support network than Spanish women have’. (MP9)

*Note*: Community of Madrid, 2019.

### 3.4 Appropriateness

Both groups of informants point out that the needs of young women are not covered since they do not find the help that they need, they feel ‘dizzy’ going from one service to another without finding a solution to help them in their process or the services are not adapted to young people. All of this poses barriers for the services to be friendly. Among young women, secondary victimization is hinted at, since their needs are not met. Therefore, they are forced to continue searching, receiving inadequate care from the system, constantly reliving their process. On the other hand, one of the professionals indicated that the deficiencies in the coverage of the needs of young women could be related to the fact that the centres are more adapted to adult women.

The lack of multidisciplinary teams made up of professionals with specific training in caring for teenagers is perceived by professionals as an important barrier that makes having friendly services difficult (Table 9).

**Table 9:** Barriers to the appropriateness of IPV services for young women

Types	Barriers
Lack of needs coverage	‘In the social security they made me dizzy going from one place to another and I felt they didn't have any interest in helping me’. (YW3); ‘…I was annoyed that nobody knew where to send me or where I could go, that nobody showed any interest in helping me, not even my family or friends, and the professional help I tried to get, well, I didn't have the strength to assimilate or verbalise what was happening to me and I just wanted to end my life but I didn't feel capable’. (YW7) ‘From my experience, in general, the centres are more adapted to care for adult women than teenage girls…. sometimes, they don't feel that is their place….’. (MP14)
Lack of multidisciplinary teams	‘…we treat adults. We don't have a child psychologist… the main services to help teens is in Madrid, this entails great difficulties for people with regard to transport, money, time…’.(MP4)

*Note*: Community of Madrid, 2019.

### 3.5 Effectiveness

Both professionals and young women stated that the scarcity of resources and the lack of adequate time to meet the needs of young women pose a barrier to the effectiveness of a friendly service. They also stated that the time delays between sessions as well as the need to devote more consultation time to adapt to the recovery processes of young women are also barriers. The budget deficit and the need for more economic resources to develop more activities are also highlighted. Likewise, professionals and young women agree on the lack of human resources and inadequate organization of the service that does not allow the provision of quality care for young women, particularly with regard to psychological care. Also, both groups interviewed reported on the lack of interpersonal communication skills and professional competencies required for IPV interventions with young women/teenagers as barriers.

Once again, they agreed that the medicalization of processes or the difficulties in finding psychological help are barriers to provision of effective services. Such medicalization refers to the fact that some professionals prescribe tranquillizers, among other medication, as a solution to the problem, instead of referring them to psychological therapy. One of the professionals related this medicalization to the lack of training of the healthcare personnel in gender perspective. Both groups of informants also agreed that this lack of training in gender perspective and in youth care acts as a barrier for the services to be effective.

On the other hand, some young women identified the lack of interpersonal skills of some professionals, the lack of empathy or active listening as barriers. They also agreed that the lack of action protocols and adherence to updated evidence‐based clinical practice guidelines adapted to young women represents a barrier to the effectiveness of the services.

On the other hand, professionals felt that, despite the existence of protocols, the lack of coordination between the different services represents a barrier, given that they use different protocols in the different services and there is no formal coordination (Table 10).

**Table 10:** Barriers to the effectiveness of intimate partner violence‐related services for young women

Types	Barriers
Resources time: enough and adequate	Time resources: ‘I didn't like the delays in time… It was directly related to the lack of resources…’ (YW2); ‘we need a bit more time because sometimes it took me a while to get started and with such little time I didn't feel I covered all the topics that I needed to in one session …’ (YW6); ‘…to do the check‐up…. vaccines and all the physical check‐up, the social part, school…so in 15 minutes we are very very limited’ (MP13). Economic resources: ‘I would have liked to have more economic resources available to be able to do workshops, activities and meetings with other women…’ (YW1); ‘budget, personnel. So it's always the same story that we don't have the cash to do many things we want to start doing… but if we do more we have to stop doing other things. So, the resources…’ (MP6). Human resources: ‘I tried to go to psychologists through the social security, but they gave me appointments every 3 months and every time I went it was a different person and I had to start from the beginning… I never got anywhere’. (YW3); ‘That's it, the psychological services of the social security, well, they are completely saturated …. We really don't have the capacity, that is, we don't have the personnel to care for people here…’ (MP9)
Competent professionals	Medicalization of the process: ‘The psychiatrist… I went once a month, went in for 5 minutes for the consultations and that's it… When she asked me how I was and I said bad, the only thing she did is increase my medication (I was taking 10–15 pills a day). I didn't experience any kind of sensation, “I looked like a zombie”, “this wasn't life” …’ (YW8); ‘…they should study medicine more from a gender perspective because pain is very medicalised, the discomfort of women is very medicalised… with all the treatments they give her…’ (MP13). Lack of training: ‘I believe this should be a struggle for all professionals, to have compulsory training, just like people are committed now and everyone has become aware of the coronavirus for the last 5 months … Why do so some people still think that violence doesn't exist?…’ (YW12); ‘…we don't have specific training to care for teenage cases…We would like training. (MP5); ‘professionals are willing to receive training; but when they are about gender or violence, well there are many biases, there is reluctance… the SERMAS (Madrid's Health Service) has put in place the figure, but they don't support nor train them… and training is carried out by ourselves, that is in many centres there are people responsible for violence, but they aren't trained …’ (MP13); ‘Therefore, one of the greatest barriers is the lack of training for professionals, of any institution …’ (MP15). Lack of interpersonal skills: ‘they need to transmit affection and understanding… It's not easy to tell, you think they're not going to believe you or they're going to think you're crazy… Active listening is needed and to take the necessary pauses’. (YW3); ‘Respect of personal time and processes. With the youth, the intensity of experiences is different… without this respect, the relationship with the professional and thus the therapy or its continuity is affected’. (YW2)
Protocols and clinical practice guidelines	‘Treatment protocols need to be established, also according to age and family situation. I think protocols are too standardised as if all women had the same burdens or family or work needs’. (YW1); ‘the coordination topic is a complicated one… we each have our own protocols, but there should be some common protocols, so all people know what they have to do…’ (MP3); ‘On the one hand social workers, lawyers on the other, and psychologists on the other…There are no formal spaces for coordination’. (MP5)

*Note*: Community of Madrid, 2019.

## 4 DISCUSSION

The results of this study point out the existence of several barriers, in the context of the study, to providing IPV care services that are considered friendly for young women. These barriers are related to the lack of information, within the social environment, about the different support services available, the scarce dissemination of these services in the community, the cost of travelling to access the services, inadequate schedules for the needs of young women, inappropriate location of the services and lack of provision of services in settings that are close to young women, such as educational centres. Barriers are also linked to the need for policies and procedures to guarantee confidentiality, settings that are unwelcoming for young women, lack of speed in providing services and/or referrals and professional malpractice attitudes. On top of these is the limitation posed by the discriminatory attitudes of some professionals towards certain population groups that already suffer social discrimination, the need for protocols to ensure equitable provision of services to young women of different social status, the lack of coverage of the needs of young women and the scarcity of multidisciplinary teams. Finally, barriers such as shortage of time and resources (financial and human), lack of competent professionals with adequate training coupled with deficiencies in interpersonal skills and lack of protocols and coordination between the different services were also highlighted.

Making resources accessible to young women is the first step towards effective IPV care in young people. As has been shown in other studies, young women first rely on informal and trusted support networks.^12^, ^18^ For them to become aware of the services that are available, it is essential to publicize in the community what these services are and what they offer, thus obtaining the support of the environment close to the young women.^19^ A study in the Swedish context, based on the WHO model in youth clinics, implemented strategies to overcome barriers similar to those that have been described by the professionals in this study.^20^ To overcome accessibility barriers, they focused on the availability of extended schedules, adapting them to the lifestyle of the youth population. Applying this measure to IPV care services would be very positive for young women. Additionally, it coincides with this study, in that a prior appointment to attend the services is not necessary. That is, the services remain open and when the young woman needs it and decides to ask for help, she can go directly. Another study supports this measure of not needing to make a prior appointment, highlighting among its results that young women value this fact positively.^21^

On the other hand, within the acceptability domain, both young women and professionals noted the lack of policies and procedures to guarantee confidentiality in the physical space of where the services are provided, that is, where they are allowed privacy to describe their situation and seek help. This is an important barrier to enable services to be accepted by young women, as found in other studies.^22^, ^23^, ^24^, ^25^

In the equity domain, we must keep in mind that socially vulnerable young women, such as women with disabilities, immigrants and ethnic minorities, have added difficulties in receiving care in these services, due to their lack of adaptation, which can magnify the social vulnerability of these young women. Although in Spain, and specifically in the Madrid region, access to these services is universal, there are other types of barriers related to gender‐based violence funding, language, social prejudices and the potential lack of support in their own social environment that adult immigrant women or women with disabilities face.^26^, ^27^ There are also other types of barriers that immigrant women (both young and old) face that should be taken into account, such as the lack of culturally appropriate services, the fact that some may not have a residence permit, lack of trust in the professionals, lack of specific training that may also imply a lack of cultural sensitivity by professionals towards young women suffering from abuse and complications in the migratory process to seek help.^27^, ^28^, ^29^

Regarding the appropriateness domain, the lack of satisfaction of some users in our study translates into an important barrier for young women to use the services again. Communication between users and professionals is needed. In this sense, previously published studies show how the self‐assessment of the services by young women improves user satisfaction.^22^, ^30^ In agreement with the proposal by Sheikh et al.,^31^ it is important to consider the requests made by young women, so that they can be taken into account in the set‐up of the services, thus better meeting their needs.

In the effectiveness domain, the barriers identified by both groups of informants were, above all, related to the scarcity of economic resources available in gender‐based violence services. A higher education level of primary care professionals as well as their familiarity with specific protocols allow for more effective secondary prevention, as they are more likely to want to research the topic.^32^, ^33^, ^34^ According to some studies,^35^, ^36^ women with a history of violence frequently use antidepressant or tranquillizer medication. Following the analysis of the interviews, young women and some professionals mentioned overmedication and the lack of psychological therapy as key points to improve the response to IPV, especially for young women. All of this is related to the training of professionals and mainly because of scarcity of psychologists in the national health system. Likewise, in some studies, the lack of training is perceived as a barrier to developing an effective health response.^32^, ^33^, ^37^ It is important for professionals to be familiar with protocols, but even more so that these protocols are standardized for the different services, and that they allow a transversal and real coordination between services so that the resources are effective. On the other hand, good communication and interpersonal skills with youth are associated with professional skills. According to Ambresin et al.^22^ and Rutherford et al.,^38^ active listening skills, clarity and interpersonal communication are also positively valued.

Among the limitations of this study is the way in which the written information was collected from the young women who survived IPV. As an in‐person interview was not carried out, it is possible that not all the information was collected, given the role that an interviewer can play during a face‐to‐face interview. That is, in an in‐person interview, the interviewer may rephrase the questions, thus gathering more information. Another limitation is that the information provided by the young women is based on their personal experiences with the formal support services that they have contacted. These experiences may vary depending on the professionals working there or the service itself. Therefore, it is possible that these results are not generalizable to other contexts, although they are applicable to some contexts with which we have compared our results. The credibility of the study was ensured with the participation of two authors (E. D. M. and C. V. C.) in the results analysis phase and the other authors' revision. The use of literal citations from the set of informants and the detailed description of the methodological process contribute towards the reliability of the study. On the other hand, the detailed description of the context and participants in the study improves the possible transferability to other settings.^39^

A considerable number of barriers related to accessibility, acceptability, equity, appropriateness and effectiveness have been identified in this study. Therefore, it is important to elaborate strategies to make changes in the IPV services in the study setting and other similar ones that can facilitate the development of youth‐friendly services for the care of young women. An increase as well as an improvement in the provision of these services are needed to raise awareness and disseminate this problem to the community, as well as to promote quality training among professionals with the aim of preventing IPV among young women, and ameliorating the care received by the young women facing it. Finally, it would be essential to incorporate the perspective of young women into the design of the care services. This would be possible by including feminist youth entities from organized civil society in the decision‐making process.

## CONFLICT OF INTERESTS

Authors declare that no competing interest exist.

## ETHICS STATEMENT

Each participant received an informed consent form, in which the objectives of the study were explained as well as the reason for the interview. Their anonymity, as well as confidentiality of the opinions they expressed, were guaranteed. Furthermore, they were assured that their participation was voluntary and that they could withdraw at any time during the interviews if they so wished. The project was approved by the Ethics Committee of the Instituto de Salud Carlos III, protocol CEI PI 61_2019‐v3.

## AUTHOR CONTRIBUTIONS

Carmen Vives‐Cases and Belen Sanz‐Barbero designed this study; Eva Durán‐Martín, Laura Otero‐García and Esther Castellanos‐Torres contributed to the acquisition and analysis of the information. All authors participated in the interpretation of data for the work; Eva Durán‐Martín and Carmen Vives‐Cases prepared the different drafts of the manuscript, integrating relevant intellectual contributions from Belen Sanz‐Barbero, Laura Otero‐García and Esther Castellanos‐Torres. All authors reviewed and approved the final version to be published, and all authors agree to be accountable for all aspects of the work in ensuring that questions related to the accuracy or integrity of any part of the work are appropriately investigated and resolved.

## Data Availability

The data that support the findings of this study are available on request from the corresponding author. The data are not publicly available due to privacy or ethical restrictions.
