# Erratum

**DOI:** 10.1111/hex.13770

**Published:** 2023-04-29

Erratum to ‘Durán‐Martín E, Vives‐Cases C, Otero‐García L, Castellanos‐Torres E, Sanz‐Barbero B. Do we have friendly services to meet the needs of young women exposed to intimate partner violence in the Madrid region? *Health Expect*. 2022;25:1058‐1068. doi:10.1111/hex.13453’

In the published article, there is an error to Eva Durán‐Martín's affiliation:

International Doctoral School, Universidad Nacional de Estudios a Distancia (UNED), Madrid, Spain

The correct affiliation is as follows:

International Doctoral School, Universidad Nacional de Educación a Distancia and Joint Research Institute of the Nacional School of Health (UNED‐IMIENS), Madrid, Spain.

We apologise for the error.

